# Surgical findings in cryptorchidism in children with Zika-related microcephaly: a case series

**DOI:** 10.1186/s12894-020-00721-3

**Published:** 2020-11-23

**Authors:** Rômulo A. L. de Vasconcelos, Ricardo A. A. Ximenes, Adriano A. Calado, Celina M. T. Martelli, Andreia V. Gonçalves, Elizabeth B. Brickley, Thalia V. B. de Araújo, Maria A. W. Rocha, Demócrito de B. Miranda-Filho

**Affiliations:** 1 Instituto de Pesquisa Aggeu Magalhães – Fiocruz, Recife, Pernambuco Brazil; 2 London School of Hygiene and Tropical Medicine, London, UK; 3 Universidade de Pernambuco, Rua Arnóbio Marques, 310 – Santo Amaro, Recife, PE 50100-130 Brazil; 4 Universidade Federal de Pernambuco, Recife, Brazil

**Keywords:** Congenital Zika syndrome, Cryptorchidism, Microcephaly, Zika virus, Orchiopexy, Surgery, Case series

## Abstract

**Background:**

Complications in the urinary tract related to congenital Zika syndrome have recently been reported. One complication, cryptorchidism, has been reported by the Microcephaly Epidemic Research Group/MERG, in Pernambuco/Brazil. The present article describes for the first time the surgical findings in a case series of boys with Zika-related microcephaly and cryptorchidism, who underwent surgical testicular exploration as a contribution to better understand the possible mechanisms involved in gonads formation and descent.

**Methods:**

A total of 7 children (11 testicular units), aged 3 to 4 years, were submitted to inguinal or scrotal orchidopexy for the treatment of palpable cryptorchidism between August 2019 and January 2020. Characteristics of the gonads and its annexes related to appendixes, testis-epididymis dissociation, gubernacular insertion, and associated hydroceles and/or hernias were described. Measures in centimetres were taken for volume calculate.

**Results:**

We found a low prevalence of testicular and epididymal appendix (66.7%), a high prevalence of testis-epididymis dissociation (55.6%), low mean testicular volume for their ages (lower for older boys) and ectopic gubernacular insertion in all cases. There was no evidence of associated hydroceles and/or hernias in any case. No surgical complication was registered or reported, and all explored gonads were properly placed in the scrotal sac.

**Conclusions:**

We herein describe the surgical findings of these children's orchidopexies and discuss the possible mechanisms of viral action in embryogenesis and postnatal growth and development of the testes and annexes. These children need to be followed over time due to the higher risk of testicular atrophy and malignancy. Surgical timing seems to be relevant to avoid loss of testicular volume.

## Background

Although neurodevelopmental impairments and structural brain defects are amongst the most common features in children with Zika-related microcephaly (ZRM), the condition has also been increasingly associated with abnormalities across a range of organ systems [1]. It is now recognized that a high frequency of children with ZRM may present with cryptorchidism [2]. Cryptorchidism is known to be associated with testicular cancer, infertility, and hypogonadism [3]. Here, we provide the first report on the findings of testicular surgical exploration in a case series of 7 male children, aged 3 to 4 years, with ZRM and cryptorchidism participating in the Microcephaly Epidemic Research Group (MERG) Paediatric Cohort, in Pernambuco, Brazil (Study Protocol: http:/www.cpqam.fiocruz.br/merg/) [4]. As main objective to better understand the possible mechanisms involved in this syndrome and its influences over gonads formation and descent.

## Methods

Between August/2019 and January/2020, a MERG paediatric urologist assessed 31 males with microcephaly (i.e., totalling 62 testicular units), using the criteria of the 2018 paediatric guidelines of the European Association of Urology, and the results have been previously described [5]. Twelve boys were diagnosed with true cryptorchidism (36.4%), while a further eleven had retractile testes (35.4%). Of the 12 boys identified as having cryptorchidism, eight underwent testicular surgical exploration. Five were operated on by a team of paediatric urologists from MERG, two by a paediatric surgeon from a private health service that was not part of the research group, and one by a paediatric surgeon at the same institution where follow-up of the MERG cohort is undertaken (NB: this child underwent surgery at age two and was subsequently recruited to the cohort, but was not included in this case series due to lack of information on surgical findings).

Laterality was defined during medical consultations by physical examination. The records regarding the aspect of the testes and annexes, gonadal volume, gubernacular insertion, and associated findings (hernias, hydroceles, and persistent peritoneal vaginal conduit) were obtained by in-surgery measurement and photographs taken by the research group during the surgical procedures. TED classification is confusing and not standardized, so it was classified in two groups: partial (head or tail attachment) or complete failure (even if attached to any structure distinct to the testis, like the gubernaculum) [6]. The location of the insertion of the gubernaculum was assessed by pulling at the gubernaculum and determining where the retraction occurred. Testicular volume was calculated using the formula: volume (cm^3^) = 0.523 × length × height × width (cm), while the volume expected for age was obtained from the formula: volume (cm^3^) = 0.004 × (age in months) + 0.438 [7]. Data on the two children who underwent surgery in the private service were obtained through telephone contact with the responsible surgeon.

All parents/guardians signed an informed consent form. This study was approved by the Oswaldo Cruz Hospital Ethical Committee (CAAE: 94544518.2.0000.5192).

## Results

Four children presented bilateral cryptorchidism with palpable gonads in the inguinal regions, two presented with palpable cryptorchidism in the left inguinal region only, and the last presented with palpable cryptorchidism in the right inguinal region only. In all cases, either inguinal or scrotal testicular exploration was performed. In one patient with bilateral cryptorchidism, exploration of the right side was not undertaken due to the occurrence of respiratory distress, which led us to curtail the surgery, following the recommendation of the anaesthesiologist. Following surgery, the child developed febrile illness, which was attributed to a respiratory viral infection, but went on to fully recover. In one of the patients with bilateral cryptorchidism submitted to surgery in the private service, exploration on the left side was not undertaken, as the attending physician decided that the surgery should be undertaken in two steps. In total, nine testes were submitted to orchidopexy. None of the children experienced complications related to surgical manipulation (Table 1).

**Table 1:** Characterization of a case series of cryptorchidism in children with ZRM submitted to surgical exploration

Patient	Age at surgery (years)	Laterality	Aspect on surgical exploration	Volume (cm^3^)	Gubernacular insertion	Associated findings
1	3	Right	Normal	Missing	Pubic	Absence
2	3	Left	TED	0.53	Pubic	TA
3	3	Bilateral	TED (both)	Right: 0.53 Left: 0.56	Pubic (both)	TA (both)
4	4	Bilateral	Right: not operated Left: TED	Right: not operated Left: 0.51	Right: not operated Left: pubic	Right: not operated Left: EA
5	3	Bilateral	Normal (both)	Right: 0.48 Left: 0.49	Pubic (both)	Right: EA Left: absence
6	4	Left	TED	0.43	Pubic	TA
7	4	Bilateral	Right: normal Left: not operated	Right: missing Left: not operated	Right: pubic Left: not operated	Right: absence Left: not operated

*TED* testis-epididymis dissociation, *TA* testicular appendix, *EA* epididymal appendix

**Fig. 1:**
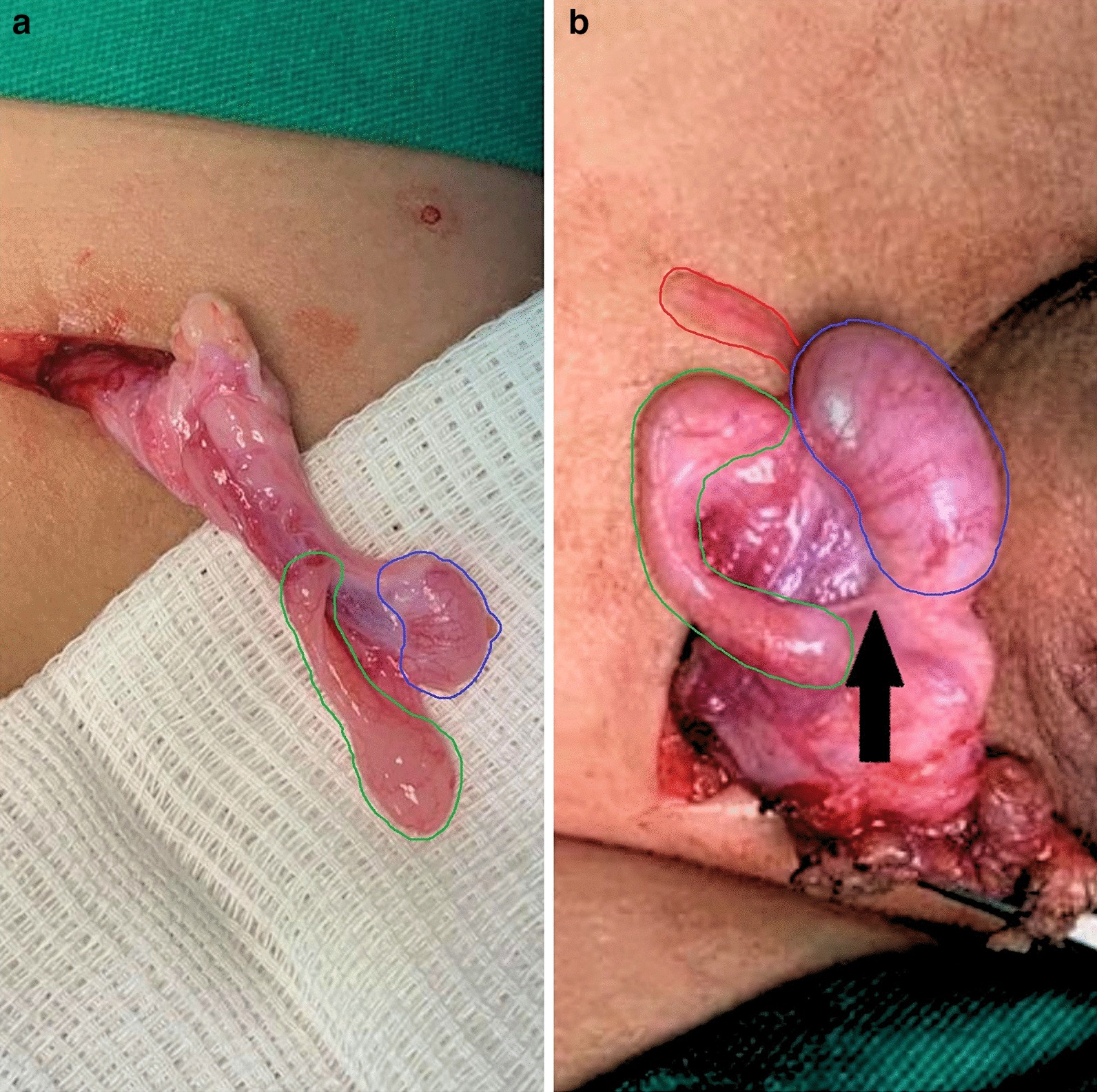
**a** TED and testicular hypotrophy; **b** TED and epididymal appendix

Five testes (55.6%) presented with testis-epididymis dissociation (TED), which was partial in one case and complete in the other four (Fig. 1). Among the testes of the children operated on 3-years of age with available data (Patients 2, 3, and 5), the mean testicular volume observed was 0.52cm^3^ (0.48–0.56cm^3^), lower than the expected mean volume of 0.60cm^3^ (0.58–0.63cm^3^). For the testes of the 4-year-old group with available data (Patients 4 and 6), the mean testicular volume was 0.47cm^3^ (0.43–0.51cm^3^), lower than the mean expected volume of 0.62cm^3^ (0.63–0.67cm^3^). In two children (Patients 1 and 7), it was not possible to obtain data for calculating testicular volume. The testicular appendix was identified in four units operated on (44.5%) and in two epididymis (22.2%). In the cases that underwent surgery by the MERG surgeons and in the two patients included from the private health service, there was no identification or reports of persistent peritoneum-vaginal canal, hydrocele, or hernia. In all five cases operated on by the MERG surgeons and in the two from the private service, the gubernaculum presented an ectopic insertion in the pubic region (Table 2).

**Table 2:** Findings of orchiopexies of a case series of cryptorchidism in children with ZRM

Mean testicular volume (n = 9 testicular units)		
3 years (5 testicular units)	0.52 cm^3^	(0.48–0.56 cm^3^)
4 years (2 testicular units)	0.47 cm^3^	(0.43–0.51 cm^3^)
Missing	2	
Testicular aspect (n = 9 testicular units)		
Normal	4	44.4%
TED	5	55.6%
Gubernacular insertion (n = 9 testicular units)		
Pubic	9	100%
Associated findings (n = 9 testicular units)		
Absence	3	33.3%
Appendix testis	4	44.5%
Appendix epididymis	2	22.2%

*TED* testis-epididymis dissociation

## Discussion

The surgical findings we presented in this paper provide additional information to our previously reported case series and enlightens the possible mechanisms related to this condition [2]. The occurrence of TED was very high in this group of boys (5 occurrences—55.6%), and was complete in 4 cases. The expected frequency of this anomaly is only 0.9% in normal children and up to 8% in patients with cryptorchidism [8]. In our series, none of the boys presented with abnormalities of the peritoneum-vaginal canal. Our children are comparable to those of cerebral palsy in relation to brain damage and high frequency of undescended testicles (36.4% in our children and 24% in children with cerebral palsy), however differ in relation to the frequency of inguinal hernias (none in our case series versus 56% in cerebral palsy) [2, 9].

Our understanding of the pathogenesis of cryptorchidism in children with ZRM is developing, and this case series provides findings that can help to better understand this physiopathologic process. Although the mechanisms of testicular descent are multi-factorial, given the high prevalence of TED and absence of anomalies of the peritoneum-vaginal conduit, it is plausible to suggest that congenital ZIKV infections should be considered as a possible explanation for the interferences on the formation mechanisms of the testis and epididymis during embryogenesis. Transmission of the Zika virus through the seminal fluid has been identified [10], which leads us to assume that there is a possible tropism of the virus to the gonadal and adnexal tissue. This possibility needs to be explored further in cadaver parts or testes submitted to orchiectomy. The frequency of gonadal appendix in our series (44.5% in the testes and 22.2% in the epididymes) was lower than the reported by Zdizvic [11], who described a frequency of 78.7% in gonads located close to the external inguinal ring in 89 normal boys. All operated testes of our series were palpable close to the external inguinal ring. Testicular appendixes are remnants of the paramesonephric ducts, but their role in testicular descent has not been well established. The function of the gubernaculum, on the other hand, is to guide the descent of the testicles in the second phase (inguinoscrotal), through the action of testosterone and the genitofemoral nerve associated with abdominal pressure, and has been well documented [12]. The finding of 100% ectopic gubernacular insertion in the pubic position could point this as being a possible mechanism responsible for the complete non-descent of these gonads and could also explain a lower, extra-abdominal location. The prevalence of ectopic gubernacular insertion in congenital cryptorchidism reported in the literature is low. Favorito [13] found no gubernacular ectopic insertion in 101 patients with cryptorchidism with a mean age of 6.4 years. Meji-de Vries [14], describing surgical findings in congenital cryptorchidism in 76 testes, found 17% of gubernacular insertion in the bottom of the scrotum and 57% in the upper scrotum, totalling 74% scrotal gubernacular insertion. Due to their location, the non-descending gonads in the children with ZRM would be subjected to thermal stress, which could contribute to a progressive loss of testicular mass and volume. In this series, children operated at three years presented with abnormally low testicular volume, and the reduction in volume was even greater in children operated at four years. Delayed surgery (i.e., beyond the recommended maximum of 18 months of age) [5] may explain the lower testicular volume in the children with ZRM.

## Conclusions

Our finding of a significant loss of testicular volume, attributed to late surgical correction, reinforces the importance of providing early attention to this anomaly. There is no apparent association with abnormalities of the peritoneum-vaginal canal, frequently seen in other children with cryptorchidism, even with cerebral palsy. The gubernaculum has an uncommon pubic insertion. A high frequency of TED calls attention to possible interference within gonadal formation, but further studies with a larger number of cases, histological examination and exploration of neuronal and hormonal abnormalities should be performed to better analyse these findings.

## Data Availability

The datasets used and/or analysed during the current study are available from the corresponding author on reasonable request.

## Abbreviations

ZRM: Zika-related microcephaly
MERG: Microcephaly Epidemic Research Group
TED: Testis-epididymis dissociation
